# Between science and transcendence: social representations of professionals about religiosity and spirituality in pediatric terminality

**DOI:** 10.1590/0034-7167-2025-0212

**Published:** 2025-11-21

**Authors:** Thamires Goulart Lambranho de Azevedo, Márcia de Assunção Ferreira

**Affiliations:** I Universidade Federal do Rio de Janeiro. Rio de Janeiro, Rio de Janeiro, Brazil

**Keywords:** Spirituality, Religion, Social Representation, Child, Health Personnel., Espiritualidad, Religión, Representación Social, Niño, Personal de Salud.

## Abstract

**Objectives:**

to analyze the social representations of religiosity and spirituality among healthcare professionals, as well as their expressions in the care provided to pediatric patients experiencing terminal illness.

**Methods:**

a qualitative, descriptive study conducted with 30 healthcare professionals at a pediatric hospital in Rio de Janeiro. Data collection was conducted through interviews and analyzed using Alceste software, based on the Theory of Social Representations.

**Results:**

participants recognized religiosity and spirituality as significant resources for comfort and coping with terminal illness. However, they reported obstacles related to institutional limitations and a lack of specific training and structure to integrate this dimension into care.

**Final Considerations:**

religiosity and spirituality constitute relevant therapeutic resources for both families and professionals. However, their effective incorporation requires specific training, institutional guidelines, and appropriate spaces to promote ethical, comprehensive, and humanized care.

## RESUMEN

**Objetivos:**

analizar las representaciones sociales sobre la religiosidad y la espiritualidad entre profesionales de la salud, así como sus expresiones en el cuidado brindado a los pacientes pediátricos que atraviesan un proceso de terminalidad.

**Métodos:**

estudio cualitativo, descriptivo, realizado con 30 profesionales de la salud en un hospital pediátrico de Río de Janeiro. La recolección de datos se llevó a cabo mediante entrevistas y fue analizada con el *software* Alceste, basándose en la Teoría de las Representaciones Sociales.

**Resultados:**

los participantes reconocieron la religiosidad y la espiritualidad como recursos significativos de consuelo y afrontamiento ante la terminalidad. Sin embargo, señalaron obstáculos relacionados con limitaciones institucionales, la falta de formación específica y la ausencia de estructura para integrar esta dimensión al cuidado.

**Consideraciones Finales:**

la religiosidad y la espiritualidad se configuran como recursos terapéuticos relevantes tanto para las familias como para los profesionales. No obstante, su incorporación efectiva requiere formación específica, directrices institucionales y espacios apropiados, con el fin de favorecer una atención ética, integral y humanizada.

## INTRODUCTION

Continuous exposure to death highlights the need to reflect on and address fears and insecurities that can interfere with healthcare professionals’ performance when caring for terminally ill patients. Terminality can be defined as the moment when all curative therapeutic possibilities have been exhausted and the patient no longer responds to established treatment, making death predictable and inevitable^(1,2)^.

When terminality occurs in childhood, it takes on even more complex dimensions, resulting in intense feelings and generating greater commotion among the healthcare team and family, due to the expectations placed on the child and their projections for their future^(1)^.

In situations of pain and suffering, such as in the care provided during terminal illness, interpersonal relationships are often redefined, whether with the family or the healthcare team. The essence of care is associated with building bonds and closeness, which find support and comfort, especially in spirituality and religion^(2)^.

Terminality is an integral part of the daily lives of healthcare professionals and, therefore, is present in their conversations and practices. However, formal training is insufficient, as the biomedical model still predominates in educational institutions and health services. This hinders the approach to the emotional, spiritual, and social aspects of human beings. Furthermore, it is important to emphasize that there is insufficient investment in the training process, both at the technical and higher education levels, to enable professionals to reflect on the feelings that emerge from the experience of terminal illness^(3)^.

Religiosity and Spirituality (R/S) are widely recognized as essential aspects in the promotion of care. However, despite the consensus on the importance of including these discussions in the training of health professionals, initiatives demonstrating their effective incorporation into curricula, including nursing, are still scarce^(4)^.

The Theory of Social Representations (TSR) is defined as a form of common-sense knowledge, directly related to how people understand and internalize information according to their own references, reinterpreting scientific knowledge according to their own convenience or according to the means and resources available^(5)^.

Social representations are socially elaborated and shared forms of knowledge that guide practices and give meaning to reality. They constitute modes of thought through which individuals appropriate an object, making it familiar and meaningful through symbolic mechanisms^(5,6)^.

## OBJECTIVES

To analyze social representations of religiosity and spirituality among healthcare professionals, as well as their expressions in the care of pediatric patients experiencing terminal illness.

## METHODS

### Ethical Aspects

The research met ethical guidelines for studies involving human subjects and was approved by the Research Ethics Committee. Data collection began after obtaining institutional authorization and all participants signed written Informed Consent Forms (ICFs). To maintain anonymity, interviews were coded according to the participants’ profession, gender, and religion, followed by a number corresponding to the interview order: Nursing Technician (NT); Doctor (MD); Nurse (Nurse); Female (F); and Male (M); religion: atheist (A), agnostic (Ag), Catholic (C), evangelical (E), Umbanda/Candomblecist (U/Cd), Kardecist (K), and no specific religion (Sre).

### Type of study

This is a qualitative, descriptive study, guided by the Theory of Social Representations (TSR), in its procedural approach, to understand the Social Representations (SR) of a group of healthcare professionals. This approach seeks to analyze how knowledge about the phenomena studied is shared and how it guides practices-in this case, healthcare^(5)^. Thus, the participants’ thought processes were investigated, exploring who holds the knowledge and where they speak (their social position), what they know, how they constructed this knowledge (processes), and with what effect (practices)^(7)^. The research report was prepared following the Consolidated Criteria for Reporting Qualitative Research (COREQ) protocol.

### Study setting

The research was conducted at a federal public institution in the city of Rio de Janeiro and involved three pediatric care units: the Pediatric Intensive Care Unit (PICU), the Pediatric Ward (PE), and the Intermediate Care Unit (UI). These units care for patients aged 29 days to 18 years, covering different levels of clinical complexity-from intensive care to mediumand low-complexity care-and have multidisciplinary teams dedicated to each department.

### Study participants

Participants were healthcare professionals from the medical and nursing teams who met the following inclusion criteria: at least one year of experience in their respective departments, providing direct and continuous care to the pediatric population. Professionals who were away from their duties, for any reason, during the data collection period were excluded from the study. The total number of professionals at the institution was 114, of which 98 met the inclusion criteria.

Participant selection followed a qualitative, intentional, and non-probabilistic sampling. The researcher initially invited 10 physicians, 10 nurses, and 10 nursing technicians working day and night shifts to participate. The study theme and objectives were presented, questions were clarified, and all invited participants agreed to participate, providing their contact information for later interview scheduling. The 30 invited participants comprised the final sample of participants, once saturation point was reached, concluding recruitment^(8)^. There were no exclusions.

### Data collection, organization, and analysis

Data collection was conducted between April and July 2024 through individual interviews, using a two-part instrument. The first part contained objective questions about the participants’ sociodemographic and professional profiles, covering profession, time since graduation, postgraduate program, religion, and how these topics were addressed during their training. The second part consisted of semi-structured questions arranged in two sections: the first addressed knowledge, experiences, and experiences regarding R/S, and the second explored the applications of this knowledge in the healthcare context. The interviews lasted an average of 20 to 40 minutes, and all were recorded and transcribed verbatim by the researcher.

The profile data were organized in Microsoft Excel 2007 spreadsheets and analyzed using simple descriptive statistics (frequency and percentage). In turn, the qualitative data were processed using lexicographic and lexicometric analysis with the aid of Alceste software (version 2012). This software applies statistical resources to count the frequency and co-occurrence of lexicons in the discourses, performing a pragmatic analysis of the communication and semantics of the discourses^(9)^. Thus, it generates dendrograms of descending hierarchical classification based on the index of statistical association of words with lexical classes, which are formed by fragments of the discourses, called Elementary Context Units (ECU), representative of the themes addressed in the discourses^(10)^.

## RESULTS

Of the 30 healthcare professionals, the majority were female (86.7%). Regarding age range, the 40-49 age group predominated (30.0%), followed by professionals between 30 and 39 years old (23.3%). 53.3% of participants reported having children.

Regarding time since graduation, half of the professionals (50.0%) had 20 or more years of experience, and the majority (86.7%) had completed some type of specialization or postgraduate degree. Regarding beliefs, 83.3% reported believing in deities, although 20.0% stated they did not follow any specific religion. The majority of participants (90% of the sample) reported having a spirituality, indicating a strong identification with this aspect, even among those who do not profess a formal religion. The main religious denominations were Kardecist Spiritism (30.0%), Catholicism (23.3%), and Evangelical Protestantism (20.0%). Other religions, such as Candomblé/Umbanda, were mentioned by 6.7% of participants, and 20.0% declared no religion.

Only 23.3% of respondents reported addressing R/S during their professional training, highlighting a significant educational gap. Despite this, 96.7% reported having experienced death in the pediatric context, highlighting the importance of preparation for dealing with subjective and existential aspects of caring for terminally ill children.

The corpus analyzed by the ALCESTE software consisted of 30 Initial Context Units (ICUs) and seven variables. In total, 81,917 occurrences of forms were identified, of which 5,187 were distinct, resulting in 1,027 forms selected for analysis after the reduction process. The average number of words per Elementary Context Unit (ECU) was 18.18.

The corpus was segmented into 1,417 ECUs, and processing continued until the data stabilized, culminating in the formation of four lexical classes. This study, however, focuses on Block 1, composed of Classes 1 and 2, which concern families’ demands for R/S-related care and how these aspects influence patient care. This block addresses discussions about Science and transcendence, highlighting tensions, contradictions, and complementarities between the two.

Class 1 highlights words related to family requests for spiritual and religious attention, as well as the conditions for meeting them. Class 2 addresses the influence of R/S in the context of care, especially in pediatric terminal situations, and also highlights strategies adopted and the challenges faced to strengthen this dimension in care practice. Table 1 lists the words most associated with lexical classes 1 and 2.

**Table 1:** Descending Hierarchical Classification Dendrogram from interviews with health professionals (N=30) from a pediatric hospital, Rio de Janeiro, Rio de Janeiro, Brazil, 2024

Lexical Class 1		Lexical Class 2	
**Family Requests**		**Influence of R/S on Care**	
**Form**	**Phi**	**Form**	**Phi**
Mother	0.37	patient	0.27
Child	0.23	care	0.27
Talk	0.21	family	0.25
Time	0.20	find	0.22
Ask	0.19	influence	0.21
Boy/girl	0.18	care	0.20
Bed	0.18	religiosity	0.18
Had	0.16	better	0.17
Child	0.15	easiness	0.17
Saw Sees	0.15 0.14	execution attention	0.16 0.15
Arrived	0.14	could	0.15
Praying	0.14	credit	0.15
Hair	0.14	comfort	0.15
Authorized	0.14	express	0.15
Stay	0.12	espirituality	0.14
Play	0.13	two	0.14
Wanting	0.12	necessity	0.13
Home	0.12	attend	0.13
Bringing	0.12	principal	0.13
Baby	0.12	affection	0.12
Baptism	0.12	profission	0.12
Death	0.12	form	0.12
Wash	0.12	impor	0.12
Father	0.11	respect	0.12
Pastor	0.11	question	0.11
Pleasuring	0.11	mortality	0.11

*Source: Report generated by Alceste software (2024).*

### Class 1: Family demands for R/S Care and institutional limitations in meeting them

Class 1 addresses family needs for R/S care and the conditions for meeting them to the extent possible, ensuring patient safety and compliance with good operating practices in health services. This Class comprises 35% of the total ECUs that make up the corpus of analysis and consists of 203 reduced forms of the full words analyzed.

In their daily care, professionals encounter different requests for religious practices and rituals that, in addition to fulfilling specific rites of certain religions, provide comfort and well-being to mothers and their families.

*At that time, parents were still considered visitors, but given the child’s terminal condition, they authorized the pastor to come. So, it was my shift; the pastor came, greeted the staff, and I took him to the bedside to pray for the child, at the family’s request.* (Nurse, F, Ser, 10)

*The mother thanked me several times because she felt the girl became calmer and more peaceful after I accepted her request and prayed for her. And, many times, mothers ask us to pray, especially when they receive a very bad diagnosis. And sometimes the mother says, “But does God want this?”.* (TE, F, E, 14)

*In fact, I spoke with the mother here once, that the child had already died, had not been baptized, and the mother came to ask me to go downstairs, to the morgue, to baptize the child, because she knew I was active in the church.* (TE, M, C, 15)

*The mother asked to put on his soccer jersey, a rosary around his neck, and began to say that her son was now free of that body, that he would run, that he would play, that he would be able to play soccer in a place without suffering and pain.* (NT, F, E, 8)

*I’m completely in favor of honoring the family’s religious requests. Some mothers see their child intubated, full of invasive devices, and are afraid to touch him, so they ask someone from the team to sprinkle holy water or anoint him with anointing oil; I always do.* (NT, F, U/Cd, 3)

*As long as there’s nothing that affects the child’s clinical condition or worsening, I’m completely in favor (of honoring the families’ religious and spiritual requests). I’ve experienced a situation like this before, and it was quite emotional.* (Nurse, F, Ser, 10)

*If the mother is nearby, the father, anyone in the family who is close by wants to hold the child, or wants to be closer, or wants to hug, or just touch, close the curtains, make the environment a little calmer.* (TE, F, E, 21)

*The child died, and the mother asked for the endotracheal tube to be removed, to remove the deep venous access. And she was allowed to help remove all the invasive devices, of course, along with the team present, because she believed the child would be free from that suffering, and we allowed her to remove the invasive devices as a way of saying goodbye. It was a team consensus. I thought it was important that the team allowed this for the mother, because it eased her pain a little. For her, helping remove the invasive devices was as if she were handing her child over to a peaceful part of life, without pain or suffering. She was relieved, and it brought her comfort.* (TE, F, E, 27)

### Class 2: Beyond the limits of science: the influence of transcendence and strategies to improve attention to R/E in care

The content of Lexical Class 2 explores the influence of R/S in the care of terminally ill children. The focus is on the need to consider transcendence in the face of the limitations of science, integrating it into the care process. Furthermore, strategies to strengthen R/S in care are discussed, promoting a more holistic and humanized approach.

Class 2 contains 42% of the total ECUs that constitute the corpus of analysis, making it the densest class. It consists of 164 reduced forms of full words found.

In the ECUs, both the influence of families’ R/S and their own beliefs on the care of terminally ill children are evident. The need to adopt a transcendent perspective on the limits of science was highlighted, especially in cases of incurable diseases, when clinical protocols are exhausted.

I strongly believe that the spirituality and religiosity of the patient and family can influence care, especially in cases of children at the end of life, because when we have a curable disease, we have a protocol, a treatment, or a scientific article to guide our practice. I think that when we’re dealing with curable diseases, this influence of the family’s religiosity and spirituality is lessened, because we have a protocol to follow, which is based on scientific evidence. (Med, F, Ser, 26)

*This comes with knowledge: the more we study, the more we can distance ourselves from our beliefs, so we can best welcome them. The influence has always been more in the matter of my own thoughts and sharing them with the patient’s family. I think we first listen and try to understand the family member and their beliefs. And we should never belittle or take away anything they might cling to and encourage. I’ve heard many doctors say, “Oh, miracles don’t exist, miracles don’t happen”. I think we can never put our beliefs above theirs, and we should encourage them to have hope based on all their beliefs, while also explaining what medicine can do and where it can go.* (Med, F, Ser, 26)

*During care, I usually pray and visualize good things, especially for children with poor prognoses, but I always do it mentally, because we have to respect that not everyone believes. And sometimes the family’s faith isn’t the same as mine.* (Nurse, F, K, 17)

*And so, even God has his limits, you know? There are things that are natural and won’t change, but I also understand that ignorance and this somewhat blind faith is the tool some families have to get through this difficult period. Having a belief, in some way, makes this process easier, which is so difficult, because children aren’t supposed to die, generally speaking. Although this is part of our daily lives, it’s not natural, and I think having something to hold on to gives you the purpose to continue working and to provide comfort, so that the child makes the transition as peacefully as possible.* (Med. F, K, 29)

*Actions that may reflect some expression of spirituality or religiosity-if I speak directly, I think I can even say I don’t have any-but indirectly, yes. For example, when you provide nighttime care and sometimes tell the child to “sleep with God”. It’s not just talk for the sake of it; it’s intended for the person to sleep with God’s blessings, for a peaceful night. And also, I don’t talk about religion or faith, because I think everyone finds their own answer, but when I realize that the family has that faith, that openness, I feel more comfortable talking about it.* (TE, F, E, 4)

*And, at the bedside, when there’s a very serious patient, I always cling to the faith that they will everything goes well and I try to visualize good things for the child while I’m taking care of them, changing a diaper or giving a bath, in those moments.* (TE, F, E, 27)

Furthermore, the importance of ensuring respect for families’ religious beliefs was emphasized, recognizing the diversity of practices and convictions as an essential aspect of humane and comprehensive care. Open dialogue on these issues was also considered crucial. Another point raised was the lack of a chaplaincy team specialized in addressing spiritual and religious needs, which hinders the integration of spiritual and religious care into clinical care, hindering a holistic approach to the patient.

*Another way I think could help improve is by having spaces-maybe not a chapel, because we don’t have the physical infrastructure-but a reception room where these mentors and religious leaders could come.* (Nurse, F, Ser, 10)

*I think implementing a chaplaincy service here at the hospital would be essential to improving attention to spirituality and religiosity in care. I think it would help professionals understand how to deal with patients, how to behave, how to meet demands. They would also understand the importance of having someone trained and qualified at the hospital to guide the team.* (Med, F, Ser, 18)

*We see that there are patients here with different religious beliefs, so I think respect is key. Here at the hospital, there could be an ecumenical chapel, open to people of all religious backgrounds, so that family members could go there to practice their religion.* (Med, F, C, 25)

*I try to reinforce parents’ hope, with the faith they already have. And, to improve attention to spirituality and religiosity in care, I believe the key is respect and conversation with families.* (Nurse, F, C, 22)

*Another strategy is to inform the family. I think I feel responsible for this, for being able to speak in a way that the layperson understands. If I can apply my technical knowledge, communicate effectively, including giving space to the family’s expressions of religiosity, I feel more comfortable, even if the outcome is bad.* (Med, F, Ser, 18)

## DISCUSSION

Families often seek to have their R/S needs incorporated into hospital care, especially during critical moments, such as the end of life for children in pediatric units. However, healthcare institutions face considerable difficulties in adequately meeting these demands^(11)^.

R/S is often neglected by healthcare teams due to a lack of academic training and specific training on how to approach and manage this topic. Furthermore, many professionals find themselves torn between maintaining a stance of scientific neutrality and the conflict between science and religion. This scenario can lead to distancing or difficulty in perceiving their own R/S experience, which compromises the integration of this dimension into care^(11)^.

Families’ demands for religious and spiritual support are diverse, ranging from the presence of religious leaders and permission for rituals to the construction of a hospital environment more sensitive to their beliefs^(12,13)^. However, the lack of institutional protocols, specific professional training, and adequate structures hinders the effective incorporation of R/S into healthcare practice^(11)^. Although healthcare professionals demonstrate individual efforts to meet such demands, the lack of institutional guidelines compromises the safety and continuity of care.

Some studies have reinforced the need for an approach that values transcendence as part of care, without necessarily linking it to institutionalized religion^(14,15)^. Spirituality, in this sense, should be understood as an existential dimension that includes the search for meaning, hope, and connection. This allows for broader, more respectful care that is adapted to the diverse experiences of families and children.

Religious coping acts protectively against emotional distress, and this effect is considered positive in research demonstrating that this type of coping promotes increased resilience, reduces symptoms of distress, and maintains emotional well-being in people with anxiety and depression, in addition to helping manage daily stresses^(16)^. A study with informal caregivers of children with leukemia showed that positive religious coping was a way of coping with care demands linked to the child’s health condition, which strengthened the authors’ hypothesis regarding the use of R/S as an indicator of physical and mental well-being^(15)^.

Religious/spiritual coping is a practice based on religion and/or spirituality as a way to provide comfort and strength to accept the child’s condition during a delicate and challenging period. This tool is a coping strategy widely used by families in pediatric terminal situations. Other studies have shown that, in most cases, this resource presents itself positively, promoting comfort, hope, and a redefinition of suffering. However, it can also manifest negatively, when the spiritual experience generates guilt, anguish, or a perception of divine punishment^(17)^. Identifying these manifestations is essential for planning appropriate interventions.

The role of healthcare professionals expands when R/S is considered part of care. This implies recognizing the influence of families’ beliefs on their decisions, understanding of the illness, and ways of coping with loss. Sensitive listening, empathic communication, and respect for religious diversity thus become essential clinical tools. The presence of faith contributes to a more collaborative relationship between the team and the family, promoting a more humanized and comprehensive experience^(18)^.

However, challenges persist, such as the lack of academic training in the subject, the fear of transgressing ethical boundaries, and the fragmentation promoted by the biomedical model, which hinder the integration of R/S into care. Some recent studies have highlighted the need for professional training, curricular reformulation, and the strengthening of interdisciplinary practices that include the spiritual dimension^(19,20)^.

One of the main strategies identified was the institutionalization of spiritual care. The creation of religious assistance committees, the regulation of visits and rituals, as well as the implementation of chaplaincies and ecumenical spaces are viable measures that ensure more organized and respectful care^(21,22)^. Collaboration with chaplains and spiritual care specialists is also essential to increase the teams’ resolution.

Transcendence, when embraced as part of the experience of illness and death, favors an approach that does not deny science, but rather expands it. Within the limits of healing, care is reinvented through attentive listening, sensitive presence, and respect for the beliefs of patients and their families. In this context, considering transcendence in terminal care broadens clinical practice by integrating subjective and spiritual dimensions into the experience of suffering, promoting more empathetic, humanized, and welcoming care^(19,20,23)^.

The ideas circulating within the group of professionals demonstrate that, in care, there are specific instances of science and belief, but that both can be articulated and integrated in child care. Science is also conceived as a welcoming strategy, offering technical care based on scientific knowledge that informs care protocols, as well as the beliefs of family members, who welcome them through the hope they sustain.

A complementary relationship between science and faith is observed in the intermediation of care. This demonstrates that people’s actions are informed by the multiple knowledge they construct throughout their lives, which they objectify in practices that express them^(7)^.

Professionals, family members, and patients think and act guided by their knowledge, of various types: scientific, philosophical, common sense, and religious, and, in the latter, by their faiths. The results identified exchanges between the faith of professionals and family members of children cared for in R/E practices, when they expressed themselves in a variety of ways, whether in meditative silence or objectively, with respect as a guide.

The results regarding the practices revealed ethics as a foundation of professional care, with attention, listening, and sensitivity standing out. Ethical sensitivity in care involves understanding the emotional states of vulnerable individuals, and caring behavior includes empathy, comfort, attentive listening, honesty, and nonjudgmental acceptance^(24)^. When it comes to R/S, freeing oneself from judgment is a prerequisite for ethical care.

In this sense, the representations identify that the concepts of Science, Faith, and Religion are interconnected in the understanding that, in the care of terminally ill children, it is possible to include R/S practices, and that professionals have the resources to do so, but that specific spaces are necessary to optimize the quality of care.

Healthcare institutions must invest in building an organizational culture that recognizes R/S as a healthcare right. The implementation of institutional guidelines, ongoing training for professionals, and the creation of spaces for spiritual practices - such as chaplaincies and welcoming environments - are fundamental measures to ensure humanized care. The integration of R/S into pediatric hospital care improves the quality of care, strengthens therapeutic bonds, and promotes a more compassionate environment in which life is valued in all its dimensions, even in the face of finitude.

### Study limitations

A limitation of this study is the fact that it was conducted in only one institution specializing in maternal and child health, which restricts a more comprehensive discussion in other contexts. Furthermore, the qualitative approach adopted does not allow for generalization of the results to other populations or care settings, an aspect that should be considered when interpreting the findings.

### Contributions to Nursing

The contributions to practice relate to training and health institutions. Training institutions should advance the inclusion and development of discussions on the topic of R/S and its influences on health and care, as already indicated in a study conducted in the palliative care field^(25)^. Research results discussed during training can inform practices that are not guided solely by individual initiatives of professionals.

## FINAL CONSIDERATIONS

The analysis of the SR on R/S showed that these dimensions emerge as fundamental in terminally ill child care, not only as emotional support for families and children themselves, but also as promoters of resilience, hope, and bonding in care. Expressions of R/S proved to be a care resource for healthcare professionals themselves, based on their own beliefs and ethical sense. However, the findings of this study highlight significant gaps in academic training. Many professionals recognize the value of R/S in coping with suffering and are guided by their own beliefs, emphasizing that ethics in care must prevail.

## Data Availability

Not applicable.
