# Supplementary material for: Between science and transcendence: social representations of professionals about religiosity and spirituality in pediatric terminality
Source: Rev Bras Enferm. 2025 Nov 21;78(Suppl 2):e20250212. doi: 10.1590/0034-7167-2025-0212 (PMC12707844; doi:10.1590/0034-7167-2025-0212)
Supplement: Translation

# Translation: Entre ciência e transcendência: representações sociais de profissionais sobre religiosidade e espiritualidade na terminalidade pediátrica

**DOI:** 10.1590/0034-7167-2025-0212pt

**Authors:** Thamires Goulart Lambranho de Azevedo, Márcia de Assunção Ferreira

**Affiliations:** I Universidade Federal do Rio de Janeiro. Rio de Janeiro, Rio de Janeiro, Brasil

**Keywords:** Espiritualidade, Religião, Representação Social, Criança, Pessoal de Saúde.

## Abstract

**Objetivos:**

analisar as representações sociais sobre religiosidade e espiritualidade entre profissionais de saúde, bem como suas expressões no cuidado prestado aos pacientes pediátricos que vivenciam o processo de terminalidade.

**Métodos:**

estudo qualitativo, descritivo, realizado com 30 profissionais de saúde em um hospital pediátrico no Rio de Janeiro. A coleta de dados foi realizada por meio de entrevistas e analisada pelo *software* Alceste, fundamentando-se na Teoria das Representações Sociais.

**Resultados:**

os participantes reconheceram a religiosidade e espiritualidade como recursos significativos de conforto e enfrentamento diante da terminalidade. Entretanto, relataram obstáculos relacionados às limitações institucionais, ausência de formação específica e estrutura para integrar essa dimensão ao cuidado.

**Considerações Finais:**

a religiosidade e a espiritualidade configuram-se como recurso terapêutico relevante tanto para as famílias quanto para os profissionais. No entanto, sua incorporação efetiva exige uma formação específica, diretrizes institucionais e espaços apropriados, de modo a favorecer um cuidado ético, integral e humanizado.

## INTRODUÇÃO

A exposição contínua à morte evidencia a necessidade de refletir e lidar com medos e inseguranças que podem interferir na atuação do profissional de saúde no cuidado ao paciente terminal. A terminalidade pode ser definida como o momento em que se esgotam todas as possibilidades terapêuticas curativas e o paciente deixa de responder ao tratamento instituído, tornando a morte previsível e inevitável^(1,2)^.

Quando acontece na infância, a terminalidade adquire contornos ainda mais complexos, resultando em sentimentos intensos e gerando maior comoção entre a equipe de saúde e a família, devido às expectativas depositadas na criança e a sua projeção para o seu futuro^(1)^.

Em situações de dor e sofrimento, como nos cuidados prestados durante a terminalidade, as relações interpessoais são frequentemente ressignificadas, seja com a família ou com a equipe de saúde. A essência do cuidado está associada à construção de vínculos e à proximidade, os quais encontram apoio e conforto, principalmente na espiritualidade e na religião^(2)^.

A terminalidade integra o cotidiano dos profissionais de saúde e, portanto, está presente em suas conversações e práticas. Entretanto, o preparo formal é insuficiente, uma vez que o modelo biomédico ainda predomina nas instituições formadoras e nos serviços de saúde, isso dificulta a abordagem dos aspectos emocionais, espirituais e sociais do ser humano. Além disso, é preciso destacar que não há investimento adequado no processo de formação, tanto de nível técnico quanto superior, que possibilite aos profissionais refletirem sobre os sentimentos que emergem da vivência da terminalidade^(3)^.

A Religiosidade e a Espiritualidade (R/E) são amplamente reconhecidas como aspectos essenciais na promoção do cuidado. No entanto, apesar do consenso sobre a importância de incluir essas discussões na formação dos profissionais de saúde, as iniciativas que comprovem sua efetiva incorporação nos currículos, incluindo o de Enfermagem, ainda são escassas^(4)^.

A Teoria das Representações Sociais (TRS) é definida como uma forma de conhecimento do senso comum, diretamente relacionada à maneira como as pessoas entendem e internalizam as informações de acordo com os referenciais que possuem, reinterpretando o saber científico, segundo sua própria conveniência ou de acordo com os meios e recursos que estão disponíveis^(5)^.

As representações sociais são formas de conhecimento socialmente elaboradas e partilhadas, as quais orientam práticas e atribuem sentido à realidade. Constituem modos de pensamento por meio dos quais os sujeitos se apropriam de um objeto, tornando-o familiar e significativo a partir de mecanismos simbólicos^(5,6)^.

## OBJETIVOS

Analisar as representações sociais sobre religiosidade e espiritualidade entre profissionais de saúde, bem como suas expressões no cuidado aos pacientes pediátricos que vivenciam o processo de terminalidade.

## MÉTODOS

### Aspectos éticos

A pesquisa atendeu às diretrizes éticas para estudos com seres humanos e foi aprovada pelo Comitê de Ética em Pesquisa. A coleta de dados foi iniciada após a autorização institucional e a assinatura do Termo de Consentimento Livre e Esclarecido (TCLE) por todos os participantes, por escrito. Para manter o anonimato das informações, as entrevistas foram codificadas conforme a profissão, sexo e religião dos participantes, seguidas de um número conforme a ordem da entrevista: Técnico de Enfermagem (TE); Médico (Md); Enfermeiro (Enf); Sexo feminino (F) e masculino (M); religião: ateu (A), agnóstico (Ag), católico (C), evangélico (E), Umbandista/Candomblecista (U/Cd), kardecista (K) e sem religião específica (Sre).

### Tipo de estudo

Trata-se de um estudo qualitativo, de caráter descritivo, cuja análise foi orientada pela Teoria das Representações Sociais (TRS), em sua abordagem processual, para compreender as Representações Sociais (RS) de um grupo de profissionais de saúde. Esta abordagem busca analisar como os saberes sobre os fenômenos estudados são compartilhados e de que maneira orientam as práticas - no caso desta pesquisa, o cuidado em saúde^(5)^. Desse modo, investigou-se o percurso do pensamento dos participantes, explorando quem detém o saber e onde essa pessoa fala (seu lugar social), sobre o que sabe, como construiu esse saber (os processos) e com que efeito (práticas)^(7)^. A elaboração do relatório de pesquisa seguiu o protocolo *Consolidated Criteria for Reporting Qualitative Research* (COREQ).

### Cenário do estudo

A investigação foi conduzida em uma instituição pública federal, situada na cidade do Rio de Janeiro, e envolveu três unidades assistenciais pediátricas: a Unidade de Terapia Intensiva Pediátrica (UTIP), a Enfermaria de Pediatria (EP) e a Unidade Intermediária (UI). Essas unidades atendem pacientes com idades entre 29 dias e 18 anos incompletos, abrangendo diferentes níveis de complexidade clínica - desde cuidados intensivos até a assistência de média e baixa complexidade -, e contam com equipes multiprofissionais específicas para cada setor.

### Participantes do estudo

Os participantes foram profissionais de saúde da equipe médica e de Enfermagem, que atenderam aos seguintes critérios de inclusão: ter, no mínimo, um ano de experiência nos respectivos setores, prestando cuidados diretos e contínuos à população pediátrica. Os profissionais que estavam afastados de suas funções, por qualquer motivo, no período de coleta de dados, foram excluídos da pesquisa. O total de profissionais da instituição foi de 114, dos quais 98 atenderam aos critérios de inclusão.

A seleção dos participantes seguiu uma amostragem qualitativa, intencional e não probabilística. Os convites foram feitos pela pesquisadora, diretamente nos setores de atuação, inicialmente a 10 médicos, 10 enfermeiros e 10 técnicos de Enfermagem, atuantes nos turnos diurno e noturno. O tema e os objetivos do estudo foram apresentados, as dúvidas foram esclarecidas e todos os convidados aceitaram participar disponibilizando seus contatos para posterior agendamento das entrevistas. Os 30 convidados compuseram a amostra final de participantes, uma vez que atingiu o ponto de saturação, encerrando-se o recrutamento^(8)^. Não houve exclusões.

### Coleta, organização e análise dos dados

A coleta de dados foi realizada entre os meses de abril e julho de 2024, por meio de entrevista individual, com a aplicação de um instrumento composto por duas partes. A primeira parte continha perguntas objetivas sobre o perfil sociodemográfico e profissional dos participantes, abrangendo profissão, tempo de formação, curso de pós-graduação, religião e a abordagem desses temas durante a formação. A segunda parte foi composta de perguntas semiestruturadas, dispostas em dois blocos: o primeiro abordava o conhecimento, as experiências e as vivências sobre R/E, e o segundo explorou as aplicações desse conhecimento no contexto do cuidado em saúde. As entrevistas tiveram duração média de 20 a 40 minutos, sendo todas gravadas e transcritas na íntegra pela pesquisadora.

Os dados do perfil foram organizados em planilhas do *Microsoft Excel* 2007 e analisados por meio de estatística descritiva simples (frequência e percentual). Por sua vez, os dados qualitativos foram processados utilizando a análise lexicográfica e lexicométrica com o auxílio do *software* Alceste (versão 2012). Este *software* aplica recursos estatísticos para a contagem da frequência e da coocorrência de léxicos nos discursos, realizando uma análise pragmática da comunicação e da semântica dos discursos^(9)^. Assim, gera dendrogramas de classificação hierárquica descendente a partir do índice de associação estatística das palavras às classes lexicais, as quais são formadas por fragmentos dos discursos, denominados de Unidades de Contexto Elementar (UCE), representativos dos temas tratados nos discursos^(10)^.

## RESULTADOS

Dos 30 profissionais de saúde, a maioria era do sexo feminino (86,7%). Em relação à faixa etária, predominou o grupo de 40 a 49 anos (30,0%), seguido pelos profissionais entre 30 e 39 anos (23,3%). A presença de filhos foi referida por 53,3% dos participantes.

Quanto ao tempo de formação, metade dos profissionais (50,0%) possuía 20 anos ou mais de experiência e a maioria (86,7%) havia concluído algum tipo de especialização ou pós-graduação. Em termos de crenças, 83,3% declararam crer em divindades, embora 20,0% tenham afirmado não seguir nenhuma religião específica. A maioria dos participantes (90% da amostra) afirmou ter espiritualidade, indicando forte identificação com esse aspecto, mesmo entre os que não professam uma religião formal.

As principais designações religiosas foram o Espiritismo Kardecista (30,0%), o Catolicismo (23,3%) e o Protestantismo Evangélico (20,0%). Outras religiões como o Candomblé/Umbanda foram referidas por 6,7% dos participantes e 20,0% declararam não ter religião.

A abordagem da R/E durante a formação profissional foi relatada por apenas 23,3% dos entrevistados, evidenciando uma lacuna formativa significativa. Apesar disso, 96,7% relataram já ter vivenciado a experiência da morte no contexto da pediatria, o que destaca a relevância do preparo para lidar com aspectos subjetivos e existenciais no cuidado de crianças em terminalidade.

O *corpus* analisado pelo *software* ALCESTE foi constituído por 30 Unidades de Contexto Inicial (UCI) e sete variáveis. Ao todo, foram identificadas 81.917 ocorrências de formas, das quais 5.187 eram distintas, resultando em 1.027 formas selecionadas para a análise após o processo de redução. A média de palavras por Unidade de Contexto Elementar (UCE) foi de 18,18.

O *corpus* foi segmentado em 1.417 UCEs, e o processamento ocorreu até a estabilização dos dados, culminando na formação de quatro classes lexicais. Este estudo, no entanto, foca no Bloco 1, composto pelas classes 1 e 2, que dizem respeito às demandas das famílias por cuidados relacionados à R/E e à forma como esses aspectos influenciam o cuidado prestado aos pacientes. Esse bloco aborda discussões sobre Ciência e transcendência, evidenciando tensões, contradições e complementaridades entre ambas.

A Classe 1 destaca vocábulos relacionados às solicitações familiares por atenção espiritual e religiosa, bem como às condições para supri-las. Já a Classe 2 aborda a influência da R/E no contexto do cuidado, especialmente em situações de terminalidade pediátrica, apontando, ainda, estratégias adotadas e os desafios enfrentados para fortalecer essa dimensão na prática assistencial. Na Tabela 1 constam as palavras de maior associação às classes lexicais 1 e 2.

**Tabela 1:** Dendrograma da Classificação Hierárquica Descendente provenientes das entrevistas com profissionais de saúde (N=30) de um hospital pediátrico, Rio de Janeiro, Rio de Janeiro, Brasil, 2024

Classe Lexical 1		Classe Lexical 2	
**Solicitações familiares**		**Influência da R/E no cuidado**	
**Forma**	**Phi**	**Forma**	**Phi**
Mãe	0,37	paciente	0,27
Criança	0,23	cuid+	0,27
fal+	0,21	famili+	0,25
Vez	0,20	ach+	0,22
ped+	0,19	influenci+	0,21
menin+	0,18	cuidados+	0,20
Leito	0,18	religiosidad+	0,18
Teve	0,16	melhor+	0,17
filh+	0,15	facilit+	0,17
vei+ ve+	0,15 0,14	execução atenção	0,16 0,15
cheg+	0,14	pod+	0,15
Orar	0,14	acredit+	0,15
cabel+	0,14	confort+	0,15
autoriz+	0,14	express+	0,15
fic+	0,12	espirituali+	0,14
jog+	0,13	Dos	0,14
Quer	0,12	necessidad+	0,13
cas+	0,12	atend+	0,13
lev+	0,12	principal	0,13
Bebê	0,12	carinh+	0,12
batiz+	0,12	profission+	0,12
falec+	0,12	form+	0,12
lave+	0,12	import+	0,12
Pai	0,11	respeit+	0,12
Pastor	0,11	quest+	0,11
pra+	0,11	mort+	0,11

*Fonte: Relatório gerado pelo software Alceste (2024).*

### Classe 1: Demandas das famílias por cuidados de R/E e as limitações das instituições para atendê-las

A Classe 1 versa sobre as necessidades das famílias por cuidados ligados à R/E e as condições para atendê-las na medida do possível, garantindo a segurança dos pacientes e o cumprimento das boas práticas de funcionamento em serviços de saúde. Esta Classe reúne 35% do total das UCEs que formam o *corpus* de análise e é constituída de 203 formas reduzidas de palavras plenas analisadas.

No cotidiano da assistência, os profissionais se deparam com diferentes solicitações de práticas e rituais religiosos que, para além de cumprirem ritos específicos de determinadas religiões, proporcionam conforto e bem-estar às mães e aos familiares.

*Naquela época, inclusive, os pais ainda eram considerados visita, mas, diante desse quadro de terminalidade da criança, autorizaram que o pastor viesse. Então, era o meu plantão; o pastor veio, cumprimentou a equipe e eu o levei até o leito para orar pela criança, a pedido da família.* (Enf, F, Ser, 10)

*A mãe me agradeceu várias vezes porque sentiu que a menina ficou mais calma e com um semblante mais tranquilo depois que eu acatei o pedido e orei por ela. E, várias vezes, as mães nos pedem para orar, principalmente quando se recebe um diagnóstico muito ruim. E, às vezes, a mãe fala: “Mas será que Deus quer isso?”.* (TE, F, E, 14)

*Inclusive, já estive conversando com a mãe aqui uma vez, que a criança já havia falecido, não era batizada, e a mãe veio pedir para que eu fosse lá embaixo, no necrotério, batizar a criança, porque sabia que eu era ativo na igreja*. (TE, M, C, 15)

*A mãe pediu para botar a roupa do time de futebol, um terço no pescoço dele e começou a falar que o filho agora estava livre daquele corpo, que ele ia correr, que ele ia brincar, que ele ia poder jogar bola em um lugar sem sofrimento e sem dor.* (TE, F, E, 8)

*Eu sou super a favor de acatar os pedidos religiosos da família. Tem mãe que vê o filho ali intubado, cheio de dispositivos invasivos, e fica com medo de tocar, e pede para alguém da equipe jogar a água benta ou passar o óleo ungido; eu sempre faço.* (TE, F, U/Cd, 3)

*Desde que não tenha nada que implique na questão do quadro clínico da criança, do agravamento, eu sou completamente a favor* [de acatar as solicitações religiosas e espirituais das famílias]. *Já vivenciei uma situação dessas, inclusive com muita emoção.* (Enf, F, Ser, 10)

*Se a mãe estiver perto, o pai, quem for da família que estiver pertinho e quiser pegar no colo, ou quiser estar mais perto, ou quiser abraçar, ou só tocar, fechar as cortinas, deixar aquele ambiente um pouco mais tranquilo.* (TE, F, E, 21)

*A criança foi a óbito e a mãe pediu para que ela retirasse o tubo endotraqueal, para retirar o acesso venoso profundo. E foi permitido que ela ajudasse a retirar todos os dispositivos invasivos, claro, junto com a equipe que estava ali, porque ela acreditava que a criança estaria livre daquele sofrimento, e a gente permitiu que ela retirasse os dispositivos invasivos da criança como uma forma de despedida. Foi um consenso da equipe. Eu achei importante a equipe ter permitido isso à mãe, porque aliviou um pouco a dor dela. Para ela, ajudar a retirar os suportes invasivos, era como se ela estivesse entregando o filho para uma parte tranquila da vida, sem dor e sem sofrimento. Ela ficou aliviada e trouxe um conforto para ela.* (TE, F, E, 27)

### Classe 2: Além dos limites da Ciência: a influência da transcendência e estratégias para melhorar a atenção à R/E no cuidado

O conteúdo da Classe Lexical 2 explora a influência da R/E no cuidado de crianças em contexto de terminalidade. O foco está na necessidade de considerar a transcendência frente às limitações da Ciência, integrando-a ao processo de cuidado. Além disso, discutem-se as estratégias para fortalecer a atenção à R/E no cuidado, promovendo uma abordagem mais holística e humanizada.

A Classe 2 possui 42% do total das UCEs que constituem o *corpus* de análise, tornando-se a classe mais densa. É constituída de 164 formas reduzidas de palavras plenas encontradas.

Nas UCEs, evidenciam-se tanto a influência da R/E das famílias quanto a influência de suas próprias crenças no cuidado das crianças em contexto de terminalidade. Apontou-se a necessidade de adotar um olhar transcendente diante dos limites da Ciência, especialmente em casos de doenças incuráveis, quando os protocolos clínicos se esgotam.

*Acredito fortemente que a espiritualidade e a religiosidade do paciente e da família podem influenciar no cuidado, principalmente nos casos de crianças em fim de vida, porque, quando a gente tem uma doença curável, a gente tem um protocolo, um tratamento ou um artigo científico para guiar a nossa prática. Eu acho que, quando a gente está lidando com doenças curáveis, essa influência da religiosidade e da espiritualidade da família é menor, porque a gente tem um protocolo a ser seguido, que é baseado em evidência científica.* (Med, F, Ser, 26)

*Isso vem com conhecimentos: quanto mais a gente estuda, mais a gente consegue se distanciar das nossas crenças, para que a gente consiga acolhê-los da melhor forma. A influência sempre foi mais nas questões dos meus pensamentos mesmo e de compartilhá-los com a família do paciente. Eu acho que a gente primeiro escuta e tenta entender o familiar e o que eles se apegam e têm de crenças. E a gente nunca deve desmerecer ou retirar qualquer coisa que eles possam se apegar e estimular. Eu já ouvi muito médico dizendo: “Ah, milagre não existe, milagres não acontecem”. Eu penso que a gente nunca pode colocar a nossa crença acima da crença deles, e devemos estimular que eles tenham a esperança baseada em todas as crenças deles, sem deixar de dizer o que a Medicina pode fazer e onde a Medicina pode chegar.* (Med, F, Ser, 26)

*Durante os cuidados, costumo fazer preces e mentalizar coisas boas, principalmente para as crianças que têm prognósticos ruins, mas sempre faço mentalmente, porque a gente tem que respeitar, nem todo mundo acredita. E, às vezes a fé da família não é a mesma que a minha.* (Enf, F, K, 17)

*E assim, até Deus tem seus limites, sabe? Têm coisas que são orgânicas e não vão mudar, mas eu entendo também que a ignorância e essa coisa da fé até um pouco cega é a ferramenta que algumas famílias têm para passarem por esse período difícil. A gente ter uma crença, de alguma forma, facilita esse processo que é tão difícil, porque não é para criança morrer, de modo geral. Embora isso faça parte do nosso cotidiano, mas não é natural, e acho que você ter alguma coisa em que se apegar te dá sentido para continuar trabalhando e para dar conforto, para que aquela criança faça a passagem da forma mais tranquila possível.* (Med. F, K, 29)

*Ações que podem refletir alguma expressão de espiritualidade ou de religiosidade, se for falar de forma direta, acho que eu até posso dizer que não tenho nenhuma, mas, de forma indireta, sim. Por exemplo, quando você faz os cuidados da noite e, às vezes, fala para a criança “dormir com Deus”. Não é um falar por falar, tem uma intenção de que a pessoa durma com as bençãos de Deus, que seja uma noite tranquila. E também, não fico falando de religião, de fé, porque eu acho que cada um encontra a sua resposta, mas quando eu percebo que a família tem aquela fé, aquela abertura, eu fico mais à vontade para conversar sobre.* (TE, F, E, 4)

*E, a beira leito, quando tem algum paciente muito grave, sempre me apego a fé de que vai dar tudo certo e tento mentalizar coisas boas para a criança durante os meus cuidados de trocar uma fralda de dar um banho, nesses momentos.* (TE, F, E, 27)

Além disso, enfatizou-se a importância de garantir o respeito pelas crenças religiosas das famílias, reconhecendo a diversidade de práticas e convicções como um aspecto essencial de um cuidado humanizado e integral. O diálogo aberto sobre essas questões também foi considerado fundamental. Outro ponto levantado foi a falta de uma equipe de capelania, especializada em lidar com as necessidades espirituais e religiosas, o que dificulta a integração do cuidado espiritual e religioso ao cuidado clínico, prejudicando a abordagem holística do paciente.

*Outra forma que eu acho que poderia ajudar a melhorar é isso, de a gente ter espaços - talvez não uma capela, porque a gente não tem estrutura física - mas se tivesse uma sala de acolhimento, onde esses mentores e líderes religiosos pudessem vir.* (Enf, F, Ser, 10)

*Acho que implementar um serviço de capelania aqui no hospital seria essencial para melhorar a atenção à espiritualidade e à religiosidade no cuidado. Penso que ia ajudar os profissionais a entenderem como lidar, como se portar, como atender às demandas. Entender, inclusive, a importância disso, tendo alguém treinado e capacitado no hospital para orientar a equipe.* (Med, F, Ser, 18)

*A gente vê que tem pacientes aqui com diferentes religiosidades, então eu acho que o respeito é o principal. Aqui no hospital poderia ter alguma capela ecumênica, que fosse aberta a pessoas de todas as orientações religiosas, para que os familiares pudessem ir a esse local para praticar a sua religiosidade.* (Med, F, C, 25)

*Eu tento reforçar a esperança dos pais, com a fé que eles já têm. E, para melhorar a atenção à espiritualidade e à religiosidade no cuidado, eu acho que o principal é o respeito e a conversa com as famílias.* (Enf, F, C, 22)

*Outra estratégia é conseguir informar a família. Acho que me sinto responsável por isso, por conseguir falar de uma forma que o leigo entenda. Se eu conseguir aplicar o meu conhecimento técnico, conseguir comunicar de uma forma eficaz, inclusive dando espaço para as manifestações de religiosidade da família, eu acho que me sinto mais confortável, mesmo se o desfecho for ruim.* (Med, F, Ser, 18)

## DISCUSSÃO

Familiares frequentemente buscam que suas necessidades de R/E sejam incorporadas ao atendimento hospitalar, especialmente em momentos críticos, como no final da vida de crianças em unidades pediátricas. No entanto, as instituições de saúde enfrentam dificuldades consideráveis ao tentar atender de forma adequada a essas demandas^(11)^.

Muitas vezes a R/E é negligenciada pelas equipes de saúde devido à falta de formação acadêmica e treinamento específico sobre como abordar e lidar com essa temática. Além disso, muitos profissionais se veem divididos entre a manutenção de uma postura de neutralidade científica o conflito entre Ciência e Religião. Esse cenário pode levar ao distanciamento ou à dificuldade de percepção de sua própria vivência de R/E, o que compromete a integração dessa dimensão no cuidado^(11)^.

As demandas das famílias por acolhimento religioso e espiritual são diversas, abrangendo desde a presença de lideranças religiosas e a permissão para rituais até a construção de um ambiente hospitalar mais sensível às suas crenças^(12,13)^. No entanto, a ausência de protocolos institucionais, de capacitação profissional específica e de estruturas adequadas dificulta a efetiva incorporação da R/E na prática assistencial^(11)^. Embora os profissionais de saúde demonstrem esforços individuais para atender tais demandas, a inexistência de diretrizes institucionais compromete a segurança e a continuidade do cuidado.

Alguns estudos reforçaram a necessidade de uma abordagem que valorize a transcendência como parte do cuidado, sem necessariamente vinculá-la à religião institucionalizada^(14,15)^. A espiritualidade, nesse sentido, deve ser compreendida como uma dimensão existencial que inclui a busca por sentido, esperança e conexão. Isso possibilita uma atenção mais ampla, respeitosa e adaptada à diversidade de experiências das famílias e das crianças.

O *coping* religioso atua de forma protetiva contra o sofrimento emocional, sendo este efeito considerado positivo em pesquisas que demonstram que este tipo de *coping* promove o aumento da resiliência, reduz os sintomas de angústia e mantêm o bem-estar emocional em pessoas com ansiedade e depressão, além de ajudar a gerir os estresses cotidianos^(16)^. Um estudo com cuidadores informais de crianças com leucemia evidenciou que o *coping* religioso positivo foi uma modalidade de enfrentamento das demandas de cuidados vinculadas à condição de saúde da criança, o que fortaleceu a hipótese dos autores sobre a utilização da R/E como indicadores de bem-estar físico e mental^(15)^.

O *coping* religioso/espiritual é uma prática que se baseia na religião e/ou espiritualidade como uma forma de proporcionar conforto e força para a aceitação da condição da criança, em um período delicado e desafiador. Esta ferramenta é uma estratégia de enfrentamento amplamente empregada pelas famílias em situações de terminalidade pediátrica. Outros estudos mostraram que, na maioria dos casos, esse recurso se apresenta de forma positiva, promovendo conforto, esperança e ressignificação do sofrimento, contudo, também pode se manifestar de forma negativa, quando a experiência espiritual gera culpa, angústia ou percepção de punição divina^(17)^. Identificar essas manifestações é fundamental para o planejamento de intervenções adequadas.

O papel dos profissionais de saúde se amplia quando se considera a R/E como parte do cuidado. Isso implica reconhecer a influência das crenças das famílias sobre suas decisões, compreensões sobre a doença e maneiras de lidar com a perda. A escuta sensível, a comunicação empática e o respeito à diversidade religiosa tornam-se, assim, ferramentas clínicas essenciais. A presença da fé contribui para uma relação mais colaborativa entre equipe e família, promovendo uma experiência mais humanizada e integral^(18)^.

Contudo, os desafios persistem, tais como a falta de formação acadêmica na temática, o receio de transgredir os limites éticos e a fragmentação promovida pelo modelo biomédico que dificultam a integração da R/E ao cuidado. Alguns estudos recentes destacaram a necessidade de capacitação dos profissionais, reformulação curricular e fortalecimento de práticas interdisciplinares que incluam a dimensão espiritual^(19,20)^.

Uma das principais estratégias apontadas foi a institucionalização do cuidado espiritual. A criação de comissões de assistência religiosa, a regulamentação de visitas e rituais, bem como a implantação de capelanias e espaços ecumênicos são medidas viáveis que garantem um cuidado mais organizado e respeitoso^(21,22)^. A colaboração com capelães e especialistas em cuidado espiritual também é essencial para ampliar a resolutividade das equipes.

A transcendência, quando acolhida como parte da experiência de adoecimento e morte, favorece uma abordagem que não nega a ciência, mas a amplia. Nos limites da cura, o cuidado se reinventa por meio da escuta atenta, da presença sensível e do respeito às crenças dos pacientes e de seus familiares. Nesse contexto, a consideração da transcendência no cuidado à terminalidade amplia a prática clínica ao integrar dimensões subjetivas e espirituais à vivência do sofrimento, promovendo uma atuação mais empática, humanizada e acolhedora^(19,20,23)^.

As ideias que circulam no pensamento do grupo de profissionais mostram que, no cuidado, há instâncias próprias da Ciência e das crenças, mas que ambas podem se articular e se integrar no cuidado à criança. A Ciência é concebida, também, como estratégia de acolhimento, quando oferece o cuidado técnico baseado nos conhecimentos científicos que instruem protocolos assistenciais, assim como a crença dos familiares, que os acolhem pela esperança que eles sustentam.

Observa-se aí uma relação de complementaridade entre a Ciência e Fé na intermediação dos cuidados, isso mostra que as ações das pessoas são instruídas pelos múltiplos saberes que elas constroem ao longo da vida, os quais objetivam em práticas que as expressam^(7)^.

Os profissionais, familiares e pacientes pensam e agem orientados por seus conhecimentos, de diferentes tipos: científico, filosófico, de senso comum e religioso, e, neste último, orientados por suas fés. Nos resultados identificaram-se intercâmbios entre a fé dos profissionais e dos familiares das crianças cuidadas nas práticas de R/E, quando elas se expressavam das mais variadas formas, seja no silêncio meditativo ou de forma objetiva, tendo o respeito como balizador.

Os resultados sobre as práticas mostraram a ética como um fundamento do cuidado dos profissionais, quando a atenção, a escuta e a sensibilidade se destacam. Ter sensibilidade ética no cuidado envolve compreender os estados emocionais das pessoas vulneráveis, e o comportamento de cuidado inclui empatia, conforto, escuta atenta, honestidade e aceitação sem julgamento^(24)^. Quando se trata de R/E, livrar-se do julgamento é condição para que o acolhimento ocorra, de forma ética.

Nesse sentido, identificam-se nas representações que os conceitos de Ciência, Fé e Religião se relacionam na compreensão de que, no cuidado à criança em situação de terminalidade, é possível incluir práticas de R/E, e que os profissionais possuem recursos para fazê-lo, mas que espaços específicos são necessários para que a qualidade da atenção possa ser otimizada.

As instituições de saúde devem investir na construção de uma cultura organizacional que reconheça a R/E como direito assistencial. A implementação de diretrizes institucionais, a formação permanente dos profissionais e a criação de espaços para as práticas espirituais - como capelanias e ambientes de acolhimento - são medidas fundamentais para garantir um cuidado humanizado. A integração da R/E no cuidado hospitalar pediátrico amplia a qualidade da assistência, fortalece os vínculos terapêuticos e promove um ambiente mais compassivo, no qual a vida é valorizada em todas as suas dimensões, mesmo diante da finitude.

### Limitações do estudo

Como limitação do estudo, considera-se o fato de ter sido realizado em apenas uma instituição especializada em saúde materno-infantil, o que restringe uma discussão mais abrangente em outros contextos situacionais. Além disso, a abordagem qualitativa adotada não permite a generalização dos resultados para outras populações ou contextos assistenciais, aspecto que deve ser considerado na interpretação dos achados.

### Contribuições para a área da Enfermagem

As contribuições para a prática são relativas às instituições de formação e de saúde. As instituições de formação devem avançar na inclusão e no desenvolvimento de debates sobre o tema da R/E e suas influências na saúde e no cuidado, como já indicado em uma pesquisa realizada em campo de cuidados paliativos^(25)^. Resultados de pesquisas debatidos na formação podem subsidiar práticas que não sejam balizadas, apenas por iniciativas individualizadas dos profissionais.

## CONSIDERAÇÕES FINAIS

A análise das RS sobre R/E mostrou que estas dimensões emergem como fundamentais no cuidado à terminalidade da criança, não apenas como suporte emocional para as famílias e as próprias crianças, mas também como promotora de resiliência, esperança e vínculo no cuidado. As expressões de R/E se mostraram um recurso de cuidado para os próprios profissionais de saúde, a partir de suas próprias crenças, bem como por senso ético. No entanto, os achados deste estudo evidenciam lacunas significativas na formação acadêmica. Muitos profissionais reconhecem o valor da R/E no enfrentamento do sofrimento e se guiam por suas próprias crenças, com destaque para que a ética no cuidado deve prevalecer.

## Data Availability

Não se aplica.
